# Mbov_0503 Encodes a Novel Cytoadhesin that Facilitates *Mycoplasma bovis* Interaction with Tight Junctions

**DOI:** 10.3390/microorganisms8020164

**Published:** 2020-01-23

**Authors:** Xifang Zhu, Yaqi Dong, Eric Baranowski, Xixi Li, Gang Zhao, Zhiyu Hao, Hui Zhang, Yingyu Chen, Changmin Hu, Huanchun Chen, Christine Citti, Aizhen Guo

**Affiliations:** 1 The State Key Laboratory of Agricultural Microbiology, College of Veterinary Medicine, Huazhong Agricultural University, Wuhan 430070, China; xifang11@hotmail.com (X.Z.); 15071362285@126.com (Y.D.); amberlixixi@163.com (X.L.); happygang@outlook.com (G.Z.); hzy13659892969@163.com (Z.H.); dkyzhanghui@163.com (H.Z.); chenyingyu@mail.hzau.edu.cn (Y.C.); hcm@mail.hzau.edu.cn (C.H.); chenhch@mail.hzau.edu.cn (H.C.); 2 Hubei International Scientific and Technological Cooperation Base of Veterinary Epidemiology, International Research Center for Animal Disease, Ministry of Science and Technology of China, Wuhan 430070, China; 3 Key Laboratory of Preventive Veterinary Medicine in Hubei Province, The Cooperative Innovation Center for Sustainable Pig Production, Wuhan 430070, China; 4 Key Laboratory of Development of Veterinary Diagnostic Products, Key Laboratory of Ruminant Bio-products, Ministry of Agriculture and Rural Affairs of China, Wuhan 430070, China; 5 IHAP, ENVT, INRAE, Université de Toulouse, Toulouse 31300, France; eric.baranowski@envt.fr (E.B.); christine.citti@envt.fr (C.C.)

**Keywords:** *Mycoplasma bovis*, transposon mutagenesis, epithelial cells, cytoadhesin, Mbov_0503, translocation, tight junction

## Abstract

Molecules contributing to microbial cytoadhesion are important virulence factors. In *Mycoplasma bovis*, a minimal bacterium but an important cattle pathogen, binding to host cells is emerging as a complex process involving a broad range of surface-exposed structures. Here, a new cytoadhesin of *M. bovis* was identified by producing a collection of individual knock-out mutants and evaluating their binding to embryonic bovine lung cells. The cytoadhesive-properties of this surface-exposed protein, which is encoded by Mbov_0503 in strain HB0801, were demonstrated at both the mycoplasma cell and protein levels using confocal microscopy and ELISA. Although Mbov_0503 disruption was only associated in *M. bovis* with a partial reduction of its binding capacity, this moderate effect was sufficient to affect *M. bovis* interaction with the host-cell tight junctions, and to reduce the translocation of this mycoplasma across epithelial cell monolayers. Besides demonstrating the capacity of *M. bovis* to disrupt tight junctions, these results identified novel properties associated with cytoadhesin that might contribute to virulence and host colonization. These findings provide new insights into the complex interplay taking place between wall-less mycoplasmas and the host-cell surface.

## 1. Introduction

Mycoplasmas are minimal, wall-less bacteria of the class *Mollicutes*, which include many pathogenic species that cause respiratory diseases, arthritis, and urogenital tract disorders in humans and a wide range of animals [1]. Despite considerable efforts, mycoplasma virulence mechanisms in are still poorly understood. Among the few exceptions are the production of ADP-ribosylating and vacuolating cytotoxin by the human pathogen Mycoplasma pneumoniae, and the release of toxic peroxide metabolites by several human and animal mycoplasma species [2,3]. Genomic studies have greatly facilitated our understanding of the structure and dynamics of mycoplasmas [4], but most of the gene functions of the accessory genome are unknown. In addition, the key factors of the interactions between these microorganisms and their hosts remain to be identified. *Mycoplasma bovis* is an emerging pathogen that affects cattle health by causing chronic diseases. This worldwide-distributed pathogen is responsible for major economic losses in the cattle industry. As for other mycoplasma species, *M. bovis* evolution was marked by a significant loss of genetic information that has left this pathogen with limited biosynthetic capabilities [5,6] and an important dependence on host cells for the acquisition of nutrients [7,8].

Adhesion to host tissues and cells is a prerequisite for bacterial colonization and virulence, and for some mycoplasma species, adhesion-deficient strains were shown to be avirulent. For example, in *Mycoplasma gallisepticum*, the lack of the cytoadherence factors GapA and CrmA or the abnormal expression of MAG_1199 and MGA_0928 after serial passages resulted in a reduced capacity in colonization and virulence [9]. In *M. pneumoniae*, the lack of adhesins P1 and P30 abolished virulence [10,11], while the abnormal expression of adhesins auxiliary proteins P40/P90, HMW1, HMW2, and HMW3 led to avirulence by preventing P1 and P30 adhesins from binding to host cells [11,12,13]. Cytoadhesins in mycoplasmas can also be involved in the uptake of nutrients, such as OppA in *Mycoplasma hominis* [14]. *M. pneumoniae* can uptake essential amino acids, cholesterol, and other nutrients by binding to host cells through its adhesion factors [15].

Although research progress on *M. bovis* adhesion has been relatively slow, some adhesion-related factors have been identified. Both a fibronectin-binding, methylenetetrahydrofolate-tRNA-(uracil-5)-methyltransferase (TrmFO) and the NADH oxidase were identified as cytoadherence factors in *M. bovis*, and their expression was found to be down-regulated in the attenuated HB0801-P150 strain [16,17]. Other *M. bovis* cytoadhesive-related factors have been reported, such as the Fructose-1,6-bisphosphate aldolase [18,19], the P26 and P27 lipoproteins [20,21], the variable surface proteins (Vsp) [22], and the α-enolase [23]. Additionally, MBOVJF4278_00255 and MBOVJF4278_00667 were found to play a role in *M. bovis* binding to bovine mammary gland epithelial cells [24]. Yet, the characterization of several *M. bovis* cytoadhesive factors was limited to data obtained from the interaction of recombinant proteins with the host cells, a simplified setting that might not grasp the overall structural complexity of the *M. bovis* cell context. One major reason for this limitation is the lack of effective genetic tools with which to perform site-directed mutagenesis in *M. bovis*. The interaction between *M. bovis* and the host is complex [25], and elucidating the *M. bovis* adhesion process to host cells requires a systematic identification of adhesion factors. More recently, bioinformatics analyses of a genome-wide transposon mutant library generated with the Swiss strain JF4278 gave rise to the identification of several new putative adhesion-related genes [24].

In the present study, a new cytoadhesin of *M. bovis* was identified by combining transposon mutagenesis to cell culture screening. This cytoadhesin, encoded by Mbov_0503, was found to contribute to the binding of *M. bovis* to host cells and to facilitate its translocation across the epithelial cell barrier.

## 2. Materials and Methods

### 2.1. Ethics Statement

Animal experiments were conducted in strict accordance with the Guide for the Care and Use of Laboratory Animals, Monitoring Committee of Hubei Province, China, and protocols were approved by the Committee on the Ethics of Animal Experiments at the College of Veterinary Medicine, Huazhong Agricultural University (agreement no. SYXK(ER) 2015-0084 issued on 31 October 2015).

### 2.2. Plasmids and DNA Constructions

Plasmid pMT85, which contains a modified version of transposon Tn*4001* (mTn), was originally developed by Zimmerman and Herrmann [26]. Plasmid pOH/P was derived from p20-1miniO/T [27] by replacing (i) the *tetM* region by the *pac* gene, encoding a puromycin N-acetyltransferase [28], and (ii) the origin of replication of *Mycoplasma agalactiae* by its counterparts in *M. bovis* strain HB0801 [6]. For the complementation of *M. bovis* mutant T4.4, a DNA fragment containing the Mbov_0503 sequence under the control of *M. agalactiae* P40 promoter was synthesized (Beijing Tianyi Huiyuan Bioscience & Technology Inc.). The synthetic DNA fragment was ligated into plasmid pOH/P at the NotI restriction site to generate plasmid pCP-T4.4. DNA constructions were verified by DNA sequencing and introduced in *M. bovis* by transformation, as previously described [27]. Plasmid pET-30a (Novagen, Darmstadt, Germany) was used for the expression of Mbov_0503 in *Escherichia coli*. A truncated version of Mbov_0503 corresponding to amino acids 47 to 548 was PCR amplified using oligonucleotide primers 0503A1/0503F2 (Table 1). Before cloning into pET-30a, TGA tryptophan codons (stop codon in *E. coli*) were changed into TGG using primers 0503A1/A2, 0503B1/B2, 0503C1/C2, 0503D1/D2, 0503E1/E2, and 0503F1/F2 (Table 1).

### 2.3. Bacteria, Cell Lines and Culture Conditions

The *M. bovis* strain HB0801 (NCBI Reference Sequence NC_018077.1) was isolated in Hubei Province, China, in 2008, and grown in a pleuropneumonia-like organisms (PPLO) medium (BD Company, Sparks, MD, USA) supplement with 10% horse serum (Hyclone, Beijing, China), as previously described [29]. When needed, gentamicin (100 µg/mL) or puromycine (10 µg/mL) was added to the medium. The *E. coli* strains DH5α and BL21 (TransGen Biotech, Beijing, China) were cultured in Luria–Bertani broth and used for DNA cloning and protein expression, respectively. RAW264.7 murine macrophages and Madin-Darby bovine kidney (MDBK) cells were purchased from the Cell Bank of the Chinese Academy of Sciences (Shanghai, China). Embryonic bovine lung (EBL) cells were kindly provided by prof. Xue Fei (Harbin Veterinary Institute). RAW264.7 cells were propagated in Roswell Park Memorial Institute (RPMI) medium. EBL and MDBK cells were cultured in minimum essential medium (MEM) and Dulbecco’s modified Eagle medium (DMEM), respectively. All media were supplemented with 10% heat-inactivated fetal bovine serum (Gibco, Grand Island, NY, USA).

### 2.4. Transposon Mutagenesis in M. bovis and Mapping of Transposon Insertion Sites

*M. bovis* knock-out mutants were generated by random transposon mutagenesis using plasmid pMT85, as previously described [30]. Briefly, late-log phase mycoplasma cultures were harvested at 10,000 × *g* for 20 min, washed twice with ice-cold Dulbecco’s phosphate-buffered saline (DPBS; Gibco), and resuspended in 0.1 M CaCl_2_. Competent cells (~10^9^ cells) were mixed with 3 μg of plasmid DNA, 10 μg of yeast tRNA, and 1 mL of 50% polyethylene glycol (PEG) 8000 (Sigma–Aldrich, St. Louis, MO, USA). After 1 min incubation, the mixture was diluted into five mL of PPLO medium and incubated for 3 h at 37 °C. Then, mycoplasma cells were washed and resuspended in 1 mL of PPLO medium and plated onto selective PPLO solid medium. After incubation at 37 °C for 3 to 7 days, single colonies were picked and grown in 1 mL of selective PPLO broth. *M. bovis* genomic DNA (gDNA) was extracted using the MiniBEST Bacteria Genomic DNA Extraction Kit (TaKaRa, Dalian, China). For each mutant, the position of the mTn insertion in the mycoplasma chromosome was determined by sequencing the junction between the *M. bovis* gDNA and the 5′- or 3′-end of the transposon using linear amplification-mediated PCR [31]. The *M. bovis* genome was fragmented by digestion with NsiI (NEB, Ipswich, Massachusetts, USA). A dsDNA fragment was synthesized by Beijing Tianyi Huiyuan Bioscience & Technology, Inc. (Beijing, China) and ligated with digested *M. bovis* genomic fragment by using T4 ligase (NEB, Ipswich, Massachusetts, USA). The primers listed in Table 1 were used to amplify the targeted fragment. The amplified PCR products were sequenced by Beijing Tianyi Huiyuan Bioscience & Technology, Inc. (Beijing, China).

### 2.5. Mycoplasma Adhesion Assay and Selection of Mutants with A Reduced Binding Capacity

RAW264.7 and EBL cells were used for mycoplasma adhesion assays. Cells were seeded in 24-well plates at a density of 10^5^ cells/well. After overnight growth, monolayers were washed with DPBS and inoculated with 10^8^ CFU of mycoplasmas in DPBS. After 1 h incubation at 37 °C, nonadherent mycoplasmas were removed by washing with DPBS, and cell monolayers were lysed with 1 mL ice-cold water. Adherent mycoplasmas were counted by plating serial dilutions onto solid PPLO medium. Each sample was tested in triplicate, and the experiment was repeated three times independently. Mutants with a reduced binding capacity to mammalian cells were selected from a collection of knock-out mutants in which nearly 200 coding sequences (CDS) were found to be disrupted.

### 2.6. Expression and Purification of rMbovP0503

HB0801 CDS Mbov_0503 was expressed in *E. coli* as a His-tagged soluble protein, hereafter referred to as rMbovP0503. Protein expression was induced with 0.8 mM isopropyl-*β*-D-thiogalactose (IPTG) for 20 h at 16 °C. Bacteria were then resuspended in binding buffer (2 mM imidazole, 20 mM Na_3_PO_4_, 500 mM NaCl; pH 7.4) and disrupted using a high-pressure homogenizer (4 °C, 1000 bar). Insoluble material was removed by centrifugation at 12,000 x g for 30 min, and rMbovP0503 was purified by nickel affinity chromatography (GE Healthcare, Piscataway, NJ, USA). Briefly, Ni-NTA agarose columns equilibrated in a binding buffer were loaded with soluble proteins. The unspecific binding was removed with a washing buffer (10% elution buffer diluted in binding buffer), and rMbovP0503 was separated from Ni-NTA agarose with an elution buffer (200 mM imidazole, 20 mM Na_3_PO_4_, 500 mM NaCl; pH 7.4). The purity of rMbovP0503 was confirmed by SDS-PAGE, and the protein concentration was determined by BCA protein assay (Thermo Fisher Scientific, Waltham, MA, USA).

### 2.7. Western and Colony Immunoblotting Analyses

A monospecific antiserum to rMbovP0503 was produced by immunizing four-week-old female BALB/c mice. Mice were primed with 100 µg of rMbovP0503 using Freund’s complete adjuvant (Sigma–Aldrich) followed by two boosters using Freund’s incomplete adjuvant (Sigma–Aldrich Corporation, St. Louis, MO, USA), each at two-week intervals. The reactivity of rMbovP0503 with specific antisera was tested by Western blotting according to a previously described procedure [16]. Briefly, rMbovP0503 was separated by 12% SDS-PAGE and transferred onto a polyvinylidene fluoride (PVDF) membrane (Millipore Corp, Billerica, MA, USA) at 100 V for 1 h. After blocking with 5% skim milk, membranes were incubated with rMbovP0503 mouse antiserum (1: 500) or HB0801 bovine antiserum [32] (1: 500) at room temperature (RT) for 2 h, followed by 1 h incubation with horseradish peroxidase (HRP)-conjugated goat anti-mouse or -bovine IgG antibodies (1:5000, SouthernBiotech, Birmingham, AL, USA). Colony immunoblotting analysis was performed by transferring mycoplasma cells onto PVDF membranes by close contact with colonies grown on agar plates. After blocking with 5% skim milk, membranes were incubated with rMbovP0503 mouse antiserum (1:250) or rVpsX rabbit antiserum [17] (1:250) at RT for 2 h, then incubated with HRP-conjugated goat anti-mouse or -rabbit IgG antibodies (1:5000, SouthernBiotech, Birmingham, AL, USA). Western and colony blots were developed by using the enhanced chemiluminescence (ECL) substrate kit (Thermo Fisher Scientific, Waltham, MA, USA).

### 2.8. Laser Scanning Confocal Microscopy

Mycoplasmas were labeled with carboxyfluorescein diacetate succinimidyl ester (CFDA-SE, Beyotime Biotechnology, Shanghai, China) for 30 min at 37 °C. EBL cell monolayers were then incubated with CFDA-SE labeled mycoplasmas in 1 mL at a multiplicity of infection (MOI) of 10^3^. For protein binding assays, EBL cell monolayers were incubated with 10 µg of rMbovP0503, either alone or preincubated with 10 µL of anti-rMbovP0503 sera or normal mouse serum as control. After 1 h incubation at 37 °C, cell monolayers were washed with DPBS, fixed with 4% neutralized paraformaldehyde for 30 min, treated with 0.5% Triton X-100 for 5 min, and blocked with 5% BSA for 2 h at 37 °C. Actin filaments and nuclei were labeled at RT with 200 mM Rhodamine phalloidin (Cytoskeleton, Denver, CO, USA) and 100 nM 4, 6-diamidino-2-phenylindole (DAPI), respectively. The binding of rMbovP0503 was visualized using rMbovP0503 mouse antiserum and Alexa Fluor^®^ 488-conjugated goat antimouse IgG (H+L) antibody (Invitrogen Corporation, Carlsbad, CA, USA). Immunofluorescence was detected with an Olympus FV1000 laser scanning confocal microscope (Olympus FV1000 and IX81, Tokyo, Japan). The experiments were repeated three times independently.

### 2.9. ELISA Binding Assays

Membrane proteins were extracted from EBL cells using a membrane protein extraction kit (BioVision, Milpitas, CA, USA), and protein concentration was determined by BCA protein assay. ELISA binding assays were performed as previously described [33], with minor modifications. The 96-well plates were coated overnight with membrane proteins or bovine serum albumin (BSA) (100 to 400 ng/well) and blocked with 5% skim milk. For *M. bovis* adhesion assay, 10^7^ CFU were added to the wells and binding was allowed for 90 min at 37°C. For rMbovP0503 adhesion assay, two-fold serial dilutions of rMbovP0503 were added to the wells. For adhesion inhibition assay, rMbovP0503 was preincubated with two-fold serial dilutions of rMbovP0503 mouse antiserum for 1 h at 37°C. The binding of *M. bovis* and rMbovP0503 to membrane proteins was revealed by using the anti-rMbovP0503 serum (1:500) or a home-made anti-*M. bovis* monoclonal antibody (1:1000) [34], respectively. HRP-conjugated goat antimouse IgG (1:5000) (Southern Biotech, Birmingham, MI, USA) was used as secondary antibody. ELISAs were developed by using 3, 3, 5, 5-tetramethylbenzidine (TMB) substrate, and absorbance at 630 nm was determined with a microplate reader (Bio-Tek, Chicago, IL, USA).

### 2.10. Transwell Monolayer Translocation Assays

MDBK cell monolayers grown on the upper chamber of a transwell insert were used for translocation assays, as described previously [35]. Briefly, MDBK cells were grown to confluency, and the integrity of the monolayers was tested by measuring the permeability to trypan blue. Transwell inserts were inoculated with *M. bovis* (10^8^ CFU/well) in the upper chamber, and the translocation of mycoplasma cells was determined by enumerating CFU in the lower chamber after 6, 12, and 24 h of incubation at 37 °C. For laser scanning confocal microscopy, MDBK cell monolayers were treated as described above, and tight junction protein ZO was labeled using mouse anti-ZO-1 polyclonal antibody and goat antimouse secondary antibody labeled with Alexa 488 [36].

### 2.11. Bioinformatic Analysis

The protein subcellular localization was predicted by PSORTb version 3.0.2 [37]. Secretory proteins that contain signal peptide but no transmembrane domain were predicted by using SignalP 4.1 [38] and TMHMM [39]. SecretomeP 2.0 was used to predict nonclassical secretory proteins having no signal peptide and no transmembrane domain [40].

### 2.12. Statistical Analysis

Statistical analysis was performed with the SPSS software package. Student’s *t*-test for unpaired two groups of continuous data and Pearson’s chi-squared test for ratio data between two different groups were used. Differences were considered to be significant when the *p* values were lower than 0.05.

## 3. Results

### 3.1. Selection of M. bovis Mutants with Reduced Binding to Host Cells

**Figure 1:**
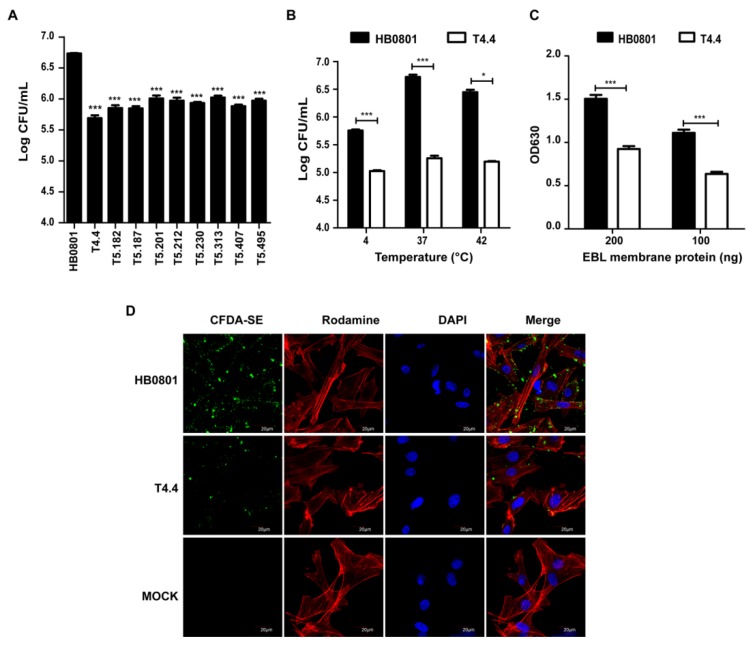
*M. bovis* mutants with altered binding properties. (**A**) Binding of *M. bovis* mutants to EBL cells. For each mutant, the binding to EBL cells was determined by counting the number of CFU associated with cell monolayers following incubation with 10^8^ CFU. Data are mean mycoplasma titers from three independent assays. Standard deviations are indicated. (**B**–**D**) Adhesion of HB0801 and Mbov_0503 knock-out mutant T4.4 to EBL cells. (**B**) Influence of the temperatures on the binding capacity. For each mycoplasma strain, the binding to EBL cells was determined by counting the number of CFU/mL associated with cell monolayers following incubation with 10^8^ CFU. Data are mean mycoplasma titers from three independent assays. Standard deviations are indicated. (**C**) Quantification of *M. bovis* binding to EBL membrane proteins. Microplates were coated with 100 ng or 200 ng EBL membrane extracts; 10^7^ CFU of each strain were used. (**D**) Visualization of CFDA-SE labeled mycoplasmas bound to EBL cells by laser scanning confocal microscopy. Actin filaments and nuclei were labeled with Rhodamine phalloidin and DAPI, respectively. PBS was used as a negative control (MOCK).

*M. bovis* mutants with reduced binding to RAW264.7 cells were selected from a collection of transposon knock-out mutants, and further tested with EBL cells. For each mutant, binding to mammalian cells was determined by counting the number of CFU associated with cell monolayers. By using this strategy, nine mutants were identified whose binding to EBL cells was 5- to 10-times lower than the parental strain (Figure 1A). Selected mutants were all characterized by the insertion of a single transposon into their chromosome that mapped within 9 CDS (Table 2). Proteins encoded by these CDS exhibited similarity to ABC transporter proteins (Mbov_0034 and Mbov_0490), tRNA modification proteins (Mbov_0059 and Mbov_0370), trigger factor (Mbov_0168), and alcohol dehydrogenase (Mbov_0353), while others were putative transmembrane proteins with unknown functions (Mbov_0154, Mbov_0305, and Mbov_0503). Of these nine proteins, five were predicted to be associated with the mycoplasma cell membrane (Mbov_0034, Mbov_0154, Mbov_0305, Mbov_0490, and Mbov_0503), and one was identified as a putative nonclassical secretory protein (Mbov_0370), a category of proteins found extracellularly, despite the absence of a signal peptide (Table 3). The remaining proteins were predicted to have a cytoplasmic localization (Mbov_0059, Mbov_0168, and Mbov_0353). Further studies are needed to confirm the role of these proteins in *M. bovis* adhesion to epithelial cells. In the present study, mutant T4.4 that displayed the most extreme phenotype with a nearly 10-fold reduction in binding to EBL cells (Figure 1A) was selected for further characterization. When compared to HB0801 that displayed optimal binding to EBL cells at 37°C, T4.4 adhesion to monolayers was only poorly affected by temperature, and was always lowers than the parental strain (Figure 1B). The reduced binding capacity of T4.4 was further confirmed by ELISA using membrane extracts of EBL cells (Figure 1C), and visualized by confocal laser scanning microscopy (Figure 1D). These data point toward Mbov_0503 product as a potential novel cytoadhesin of *M. bovis*.

### 3.2. Mbov_0503 Encodes a Novel Cytoadhesin of M. bovis

**Figure 2:**
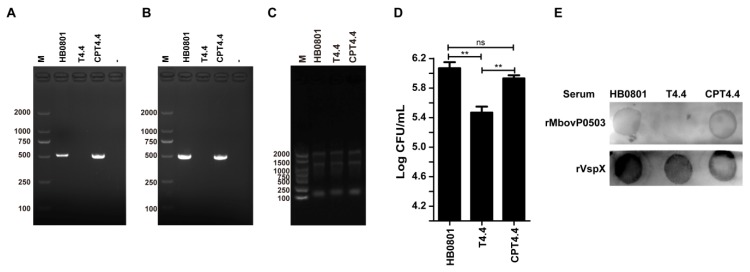
Complementation studies with Mbov_0503 knock-out mutant. (**A**) PCR amplification of Mbov_0503 in the parental strain (HB0801), Mbov_0503 knock-out mutant (T4.4), and complemented strain (CPT4.4), (**B**) RT-PCR amplification of Mbov_0503 transcripts, (**C**) electrophoretic analysis of total RNA, and (**D)** mycoplasma binding to EBL cells. Data are the means of three independent assays. Standard deviations are indicated by error bars. *P* values are indicated by asterisks (** *p* < 0.01; ns = *p* > 0.05). (**E**) Immunodetection of Mbov_0503 products at the mycoplasma surface. Colonies were incubated with rMbovP0503 or rVspX antisera.

Complementation studies were performed with mutant T4.4 to confirm the role of Mbov_0503 in *M. bovis* adhesion to EBL cells. Transformation of T4.4 with plasmid pCP-T4.4 led to the selection of the complemented strain CPT4.4 (Figure 2A). The expression of Mbov_0503 in CPT4.4 was confirmed by RT-PCR amplifications (Figure 2B) and colony immunoblotting assays (Figure 2E) that further demonstrated that Mbov_0503 product is exposed at the surface of *M. bovis* cells. As expected, no expression of Mbov_0503 was detected in T4.4 (Figure 2). Finally, plasmid pCP-T4.4 was able to restore the binding capacity of the complemented strain CPT4.4 to wild-type values (Figure 2D), ruling out any possible downstream effect of the transposon in T4.4 or the selection of a particular phase variant with altered adhesive properties. This result demonstrates that the Mbov_0503 product is surface exposed, and that it contributes to *M. bovis* adhesion to EBL cells.

**Figure 3:**
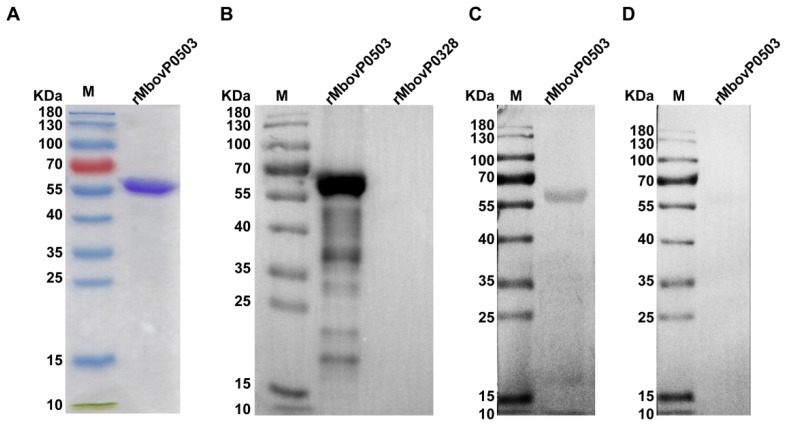
Immunoreactivity of rMbovP0503. (**A**) SDS-PAGE analysis of purified rMbovP0503. (**B**) Western blotting analysis of rMbovP0503 reactivity with the rMbovP0503 antiserum, (**C**) cattle antiserum against HB0801, and (**D**) negative cattle serum. The unrelated *M. bovis* recombinant protein rMbovP328 was used as a negative control. M: prestained protein ladder.

To further evaluate the role of Mbov_0503 in *M. bovis* adhesion to EBL cells, a truncated version of Mbov_0503 was expressed in *E. coli* by deleting the N-terminal region, which is predicted to contain a transmembrane domain (see Materials and Methods). The recombinant protein, designated as rMbovP0503, was successfully expressed in *E. coli* as a 59.4 kDa soluble protein (Figure 3A). Western blotting assays showed that rMbovP0503 was recognized by anti-rMbovP0503 antibodies (Figure 3B) and a bovine immune serum from animals experimentally infected with HB0801 (Figure 3C). As expected, the unrelated *M. bovis* recombinant protein rMbovP0328 (unpublished materials) failed to react with the rMbovP0503 antiserum (Figure 3B) and no reactivity could be observed with the negative bovine serum (Figure 3D).

**Figure 4:**
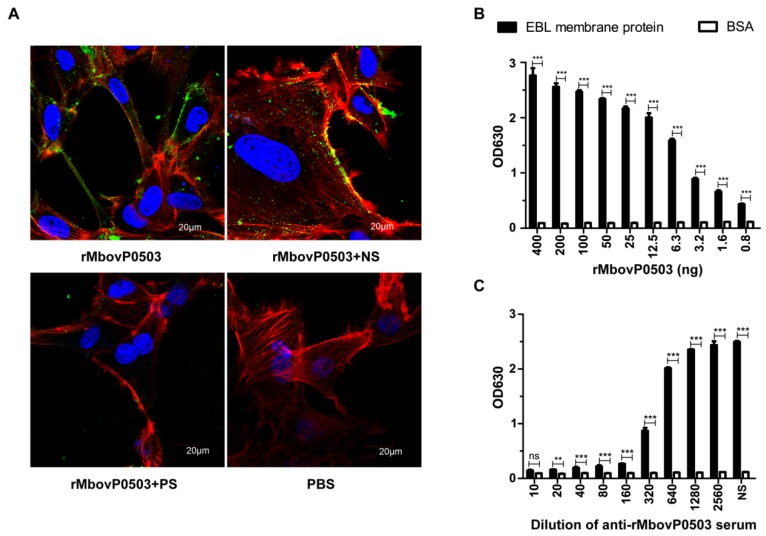
Adhesion of rMbovP0503 to EBL cells. (**A**) Visualization of rMbovP0503 bound to EBL cells by laser scanning confocal microscopy. EBL cells were incubated with 10 μg rMbovP0503 (rMbovP0503). PBS was used as a negative control. For the adhesion inhibition assay, rMbovP0503 was preincubated with rMbovP0503 antiserum (rMbovP0503 + PS) or negative serum (rMbovP0503 + NS). Proteins were probed with anti-rMbovP0503 antibodies and Alexia 488-conjugated anti-IgG antibodies (Green). Actin filaments and nuclei were labeled with Rhodamine phalloidin (Red) and DAPI (Blue), respectively. (**B**) Dose-dependent binding of rMbovP0503 to EBL membrane extracts. ELISA plates coated with 400 ng EBL membrane extracts or 400 ng BSA were incubated with serial dilutions of rMbovP0503. (**C**) Inhibition of rMbovP0503 binding to EBL membrane extracts by rMbovP0503 antiserum. rMbovP0503 (400 ng) was preincubated with serial dilutions of rMbovP0503 antiserum before incubation with EBL membrane extracts or BSA. *P* values are indicated by asterisks (*** *p* < 0.001; ** *p* < 0.01; ns = *p* > 0.05).

Adhesion of rMbovP0503 to EBL cells was visualized by laser scanning confocal microscopy. The binding of rMbovP0503 to EBL cells was significantly inhibited upon preincubation with anti-rMbovP0503 antibodies, whereas mouse negative serum did not display an obvious inhibitory effect (Figure 4A). The interaction of rMbovP0503 with membrane extracts of EBL cells was further analyzed by ELISA. When compared to BSA, rMbovP0503 was found to bind cell membrane proteins in a dose-dependent manner within a range of 0.8 to 400 ng (Figure 4B). This binding was inhibited by anti-rMbovP0503 antibodies in a concentration-dependent manner (Figure 4C). Serum dilutions required to reach 60% and 100% reduction in binding corresponded respectively to 1:320 and 1:10. These data suggest a direct interaction of Mbov_0503 product with host cells, and identify this surface protein as a novel cytoadhesin of *M. bovis*.

### 3.3. Mbov_0503 Facilitates M. bovis Interaction with Tight Junctions

**Figure 5:**
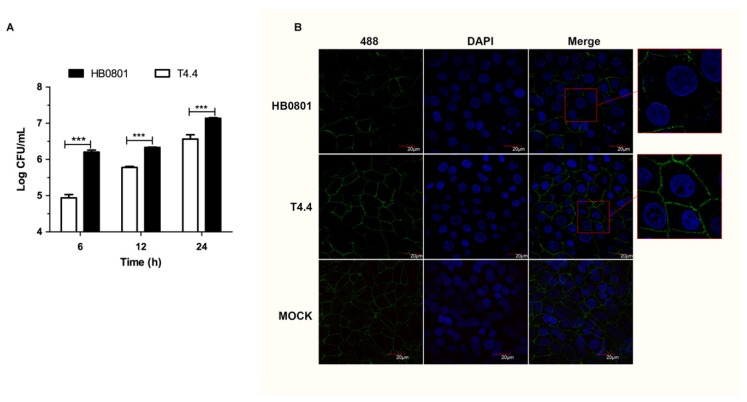
Translocation of Mbov_0503 knock-out mutant across MDBK epithelial cell monolayers. (**A**) MDBK cell monolayers grown on Transwell chambers were infected with Mbov_0503 knock-out mutant (T4.4) or the parental strain (HB0801) at the apical side, and mycoplasmas that translocated to the basal chamber were enumerated by counting CFU. (**B**) Structural integrity of the tight junctions in MDBK cell monolayers infected with the Mbov_0503 knock-out mutant (T4.4) or the parental strain (HB0801). The tight junctions were visualized by laser scanning confocal microscopy using anti-ZO-1 and Alexia 488-conjugated anti-IgG antibodies (Green). The nuclei were labeled with DAPI (Blue). MOCK-infected MDBK cell monolayers were used as a negative control (MOCK). Images in red boxes were magnified for 2.5 times. *P* values are indicated by asterisks (*** *p* < 0.001).

Mbov_0503 is not essential for *M. bovis* adhesion to host cells, and the disruption of this CDS is only associated with a partial reduction of its binding capacity. This prompted us to question the biological implications of Mbov_0503 disruption in *M. bovis*. Here, we tested the capacity of *M. bovis* to translocate across the epithelial cell barrier. Translocation assays carried out with HB0801 revealed that *M. bovis* was able to disseminate across MDBK cell monolayers (Figure 5A). Interestingly, translocation of mutant T4.4 across cell monolayers was significantly reduced at 6, 12, and 24 h postinoculation when compared to the parental strain (*p* < 0.001, student’s *t*-test). Growth curve experiments carried out with HB0801 and T4.4 failed to reveal any obvious influence of Mbov_0503 on mycoplasma proliferation (Figure S1).

To further understand the influence of Mbov_0503 on *M. bovis* translocation, laser scanning microscopy was used to visualize the integrity of tight junctions in MDBK cell monolayers. Remarkably, a massive breakdown of tight junctions was observed upon incubation with HB0801, affecting up to 70% of epithelial cells (27/39), as determined by a reduced ZO-1 immunoreactivity at the cell-cell borders (Figure 5B). In contrast, this value was only 44% (22/50) with T4.4. The difference between these two groups was statistically significant (*p* < 0.001, Pearson’s chi-squared test). Besides demonstrating the capacity of *M. bovis* to translocate across epithelial cell monolayers, these results suggest that translocation may be influenced by mycoplasma binding capacity, and thus, Mbov_0503 may facilitate *M. bovis* interaction with tight junctions by securing mycoplasma adhesion to epithelial cell monolayers.

## 4. Discussion

Because of the absence of a cell wall, membrane proteins play a critical role in the interplay between mycoplasmas and their host. These proteins are involved in multiple biological functions including adhesion, signal transduction, the uptake of nutrients, and the export of toxic metabolites [41,42,43]. Membrane proteins can also act as virulence factors, and are critical for escaping and modulating the host immune response [7,44]. In the present study, we identified a new membrane protein of *M. bovis* which is involved in cytoadhesion and facilitates the interaction of the pathogen with tight junctions.

The new cytoadhesin encoded by Mbov_0503 was identified by the screening of a collection of knock-out mutants in cell culture. The cytoadhesive properties of this protein was confirmed by ELISA and laser confocal microscopy using either a recombinant version of the cytoadhesin or the Mbov_0503 knock-out mutant. Complementation studies also rule out any influence of the transposon on other CDS located downstream of its insertion site, as well as the selection of a particular phase variant with altered binding properties. Indeed, *M. bovis* Vsp are known to undergo high-frequency phase and size variations [45], and several Vsp, including VspA, B, E, and F, have been shown to perform an important role in mycoplasma adhesion [22,46].

In mycoplasmas, cytoadhesins are often involved in several key biological functions. Beside their crucial roles in cell adhesion, they are also important for invasion, colonization, and dissemination within the hosts [47]. This prompted us to test the translocation of strain HB0801 across epithelial cell monolayers. Interestingly, the surface expression of Mbov_0503 was found to influence the interaction of *M. bovis* with tight junctions and its translocation across the epithelial barrier. Tight junctions are targeted by many pathogens, since these structures are important in sealing epithelia to form a virtually impermeable barrier to fluids and microbes [48]. Whether the Mbov_0503 product interacts directly with epithelial tight junctions is unknown, but this cytoadhesin may facilitate the interaction of *M. bovis* with these structures by enhancing mycoplasma binding to the host cell surface. Whether this newly-identified cytoadhesin influences the pathogenicity of *M. bovis* remains to be investigated. However, in the absence of a small animal model, cell culture assays provided a convenient screening system for the identification of new cytoadhesins in the cattle pathogen *M. bovis*.

In addition to Mbov_0503, *M. bovis* mutants with reduced binding to EBL cells identified several other CDS products which are potentially involved in cytoadhesion. These included proteins with similarity to the trigger factor (Mbov_0168) and the oligopeptide ABC transporter ATP-binding protein oppD (Mbov_0034), which were previously reported to be adhesion-related genes in *Streptococcus suis* and *Vibrio alginolyticus*, respectively [49,50]. Mbov_0490 is another CDS potentially related with *M. bovis* cytoadhesion. This CDS product is homologous to proteins of the ATP-binding cassette subfamily B that belong to ABC transporters, a large protein family whose members are often involved in virulence and drug resistance in bacteria [51]. Finally, Mbov_0370 was predicted to encode a nonclassical secretory protein. A number of nonclassical secretory proteins have been identified in mycoplasmas, such as EF-Tu and DnaK in *M. ovipneumoniae* [52]. Several of these proteins can have moonlighting activities, such as the EF-Tu in *M. hypneumoniae* [53] and the TrmFO and NADH oxidase in *M. bovis* that were identified to be cytoadherence factors, [16,17]. Additional studies are needed to characterize these proteins and their potential role in *M. bovis* pathogenesis.

In conclusion, the present study identified a novel cytoadhesin of *M. bovis* and a number of potential adhesion-related factors. Since binding to host cells plays a predominant role in virulence, our findings may contribute to a better understanding of these minimal pathogens, and provide interesting directions for their control.

## Figures and Tables

**Table 1:** Oligonucleotide primers used in the present study ^a^.

Primer	Oligonucleotide sequence (5′→3′)	Purpose
mTn For 1	GACCTACACCGAACTGAGATACC	Mapping of mTn insertions
Link Rev 1	CCAGTGTGCTGGAATTGCCC	
mTn For 2	CCTGCGTTATCCCCTGATTCTG	
Link Rev 2	GCAGATATCCATCACACTGGCG	
mTn Seq 3	CCTTTGAGTGAGCTGATACCGCTC	
0503A1	CGCGGATCCTTCAAAATTAATTTAGAAAAGAAAAATGTAATTAG	Mbov_0503 cloning
0503A2	TATAGTTAGGCGTAAAGCT[C]CAGTATATA	Mutagenesis
0503B1	TTACGCCTAACTATAATGAAAAAAATATG	
0503B2	CATCTTTAAAATAATA[C]CAGCCTGGGT	
0503C1	TTAAAGATGGCATTTTACACTTAATAATTG[G]	
0503C2	TTACTGTCTAAAAA[C]CACTGATAATACTTTG	
0503D1	TTTAGACAGTAATGAGCATAACGAACAC	
0503D2	TTATATAAATATCTAAA[C]CAAGGGACATTT	
0503E1	AGATATTTATATAATAATTTATG[G]ACCGAAAATT	
0503E2	CAGTTGAAAGTAAATTTTCGGT[C]CATAAATTATT	
0503F1	CTTTCAACTGAAGTAAACAGTGATGATT	
0503F2	CCGCTCGAGTTATACATTATAAAAATATTTTATTTTTAATCTTTGTACA	Mbov_0503 cloning
Mu0503F	CTCCAATACTCTAAGTGGTCTAAGT	PCR amplification of Mbov_0503
Mu0503R	CATCTTTAAAATAATATCAGCCTGGGT	

^a^ The restriction sites NcoI and XhoI are underlined; Nucleotide substitutions are in brackets.

**Table 2:** Transposon insertion sites in *M. bovis* mutants with reduced binding to EBL cells

Genomic Position ^a^	CDS ^b^	Relative CDS Position ^c^	Mutant ^d^	Predicted CDS Identity
035818 (-)	Mbov_0034	0.07 (+)	T5.407	oligopeptide ABC transporter ATP-binding protein (*oppD*)
068606 (+)	Mbov_0059	0.99 (-)	T5.182	tRNA modification GTPase (*trmE*)
175200 (-)	Mbov_0154	0.61 (-)	T5.187	transmembrane protein
191067 (+)	Mbov_0168	0.31 (-)	T5.201	trigger factor (*tig*)
359714 (-)	Mbov_0305	0.41 (+)	T5.230	transmembrane protein
420981 (+)	Mbov_0353	0.45 (+)	T5.212	alcohol dehydrogenase (*adh*)
436842 (+)	Mbov_0370	0.38 (+)	T5.313	tRNA-methyltransferase (*trmU*)
573330 (+)	Mbov_0490	0.97 (+)	T5.495	ATP-binding cassette subfamily B
586897 (-)	Mbov_0503	0.23 (-)	T4.4	transmembrane protein

^a^ Transposon insertion sites were defined based on the published HB0801 sequence (GenBank accession number CP002058). The orientation of the mTn is indicated in parenthesis. ^b^ CDS are designated according to the nomenclature used in GenBank. ^c^ The relative position and orientation of the mTn are indicated for each CDS. ^d^ Mutants were designated according to transformation and clone numbers.

**Table 3:** Predicted subcellular localization of selected *M. bovis* CDS products.

CDS	Signal Peptide ^a^	Transmembrane Domain ^a^	SecP Score ^c^	Subcellular Localization (Score) ^d^
Mbov_0034	No	No	0.800	CM (9.99)
Mbov_0059	No	No	-	C (9.97)
Mbov_0154	No	Yes	0.918	unknown
Mbov_0168	No	No	-	C (7.5)
Mbov_0305	No	Yes	0.512	C (7.5)
Mbov_0353	No	No	-	C (9.97)
Mbov_0370	No	No	0.593	C (9.97)
Mbov_0490	Yes	Yes	0.926	CM (10)
Mbov_0503	No	Yes	0.657	unknown

^a^ SignalP 4.1 prediction. ^b^ TMHMM prediction. ^c^ The SecP score determined by using SecretomeP 2.0. ^d^ PSORTb prediction; C, cytoplasm; CM, cytoplasmic membrane

## Supplementary Materials

The following are available online at https://www.mdpi.com/2076-2607/8/2/164/s1, Figure S1: Growth curve of *M. bovis*.
