# Supplementary figures and images for: Mbov_0503 Encodes a Novel Cytoadhesin that Facilitates Mycoplasma bovis Interaction with Tight Junctions
Source: Microorganisms. 2020 Jan 23;8(2):164. doi: 10.3390/microorganisms8020164 (PMC7074692; doi:10.3390/microorganisms8020164)

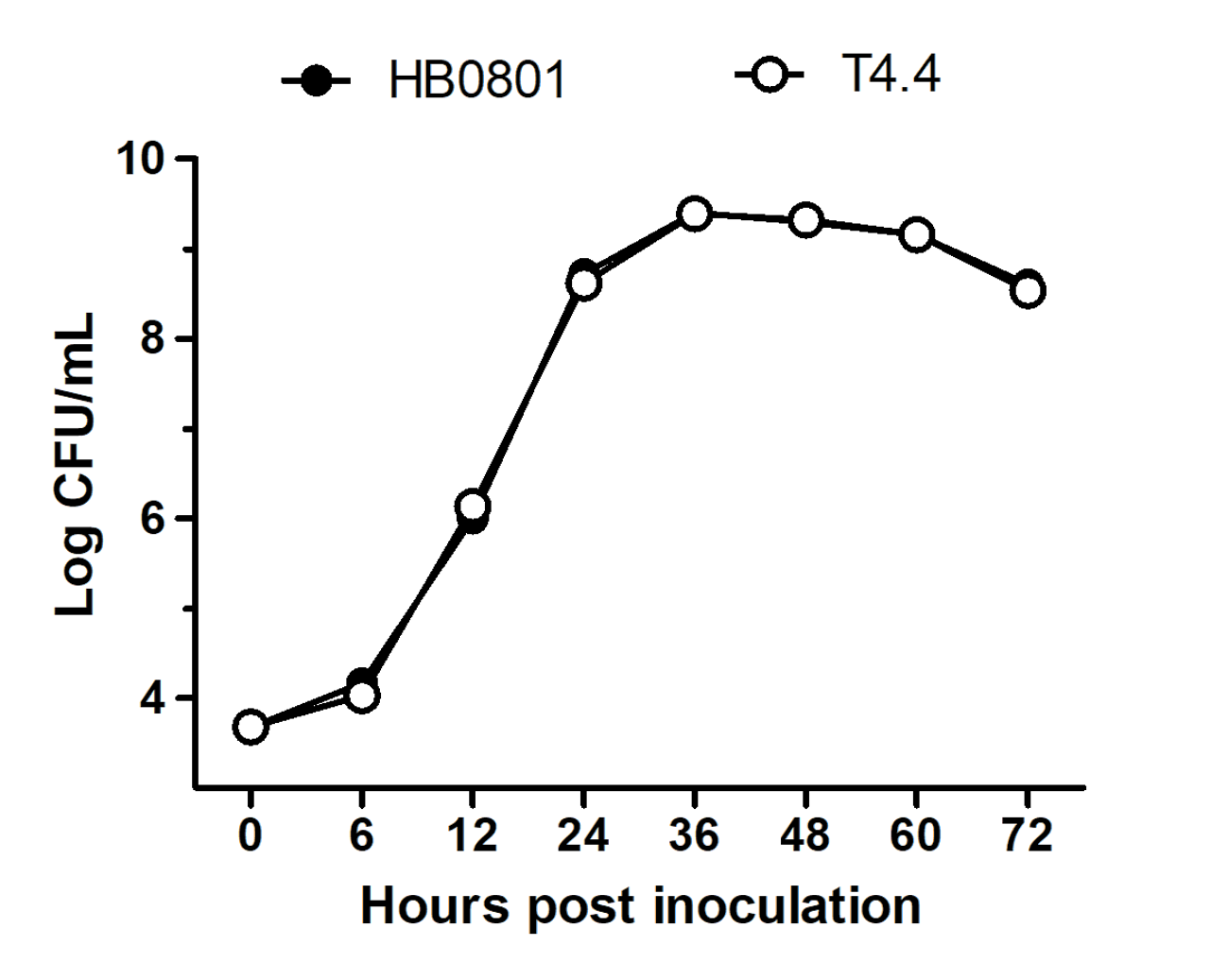

Supplement: Supplementary file 1 [file microorganisms-08-00164-s001.zip › Figure S1.tif]
